# Using the C14:1/Medium-Chain Acylcarnitine Ratio Instead of C14:1 to Reduce False-Positive Results for Very-Long-Chain Acyl-CoA Dehydrogenase Deficiency in Newborn Screening in Japan

**DOI:** 10.3390/ijns10010015

**Published:** 2024-02-20

**Authors:** Go Tajima, Junko Aisaki, Keiichi Hara, Miyuki Tsumura, Reiko Kagawa, Fumiaki Sakura, Hideo Sasai, Miori Yuasa, Yosuke Shigematsu, Satoshi Okada

**Affiliations:** 1 Division of Neonatal Screening, Research Institute, National Center for Child Health and Development, 2-10-1 Okura, Setagaya-ku, Tokyo 157-8535, Japan; aisaki-j@ncchd.go.jp; 2 Department of Pediatrics, Hiroshima University Graduate School of Biomedical and Health Sciences, 1-2-3 Kasumi, Minami-ku, Hiroshima 734-8551, Japan; hara-keiichi@kxd.biglobe.ne.jp (K.H.); m055@hiroshima-u.ac.jp (M.T.); rekagawa@hiroshima-u.ac.jp (R.K.); fsakura@kazusa.or.jp (F.S.); sokada@hiroshima-u.ac.jp (S.O.); 3 Department of Pediatrics, National Hospital Organization Kure Medical Center and Chugoku Cancer Center, 3-1 Aoyama-cho, Kure 737-0023, Japan; 4 Department of Technology Development, Kazusa DNA Research Institute, 2-6-7 Kazusa-kamatari, Kisarazu 292-0818, Japan; 5 Department of Early Diagnosis and Preventive Medicine for Rare Intractable Pediatric Diseases, Graduate School of Medicine, Gifu University, 1-1 Yanagido, Gifu 501-1194, Japan; sasai.hideo.f1@f.gifu-u.ac.jp; 6 Department of Pediatrics, Faculty of Medical Sciences, University of Fukui, 23-3 Matsuoka-Shimoaizuki, Eiheiji-cho 910-1193, Japan; miori@u-fukui.ac.jp (M.Y.); yosuke3321181@gmail.com (Y.S.)

**Keywords:** very-long-chain acyl-CoA dehydrogenase, *ACADVL*, newborn screening, false-positive, tetradecenoylcarnitine (C14:1), decanoylcarnitine (C10), octanoylcarnitine (C8), hexanoylcarnitine (C6), acylcarnitine ratio

## Abstract

Very-long-chain acyl-CoA dehydrogenase (VLCAD) deficiency is a long-chain fatty acid oxidation disorder that manifests as either a severe phenotype associated with cardiomyopathy, a hypoglycemic phenotype, or a myopathic phenotype. As the hypoglycemic phenotype can cause sudden infant death, VLCAD deficiency is included in newborn screening (NBS) panels in many countries. The tetradecenoylcarnitine (C14:1) level in dried blood specimens is commonly used as a primary marker for VLCAD deficiency in NBS panels. Its ratio to acetylcarnitine (C2) and various other acylcarnitines is used as secondary markers. In Japan, tandem mass spectrometry-based NBS, initially launched as a pilot study in 1997, was introduced to the nationwide NBS program in 2013. In the present study, we evaluated levels of acylcarnitine with various chain lengths (C18 to C2), free carnitine, and their ratios in 175 infants who tested positive for VLCAD deficiency with C14:1 and C14:1/C2 ratios. Our analyses indicated that the ratios of C14:1 to medium-chain acylcarnitines (C10, C8, and C6) were the most effective markers in reducing false-positive rates. Their use with appropriate cutoffs is expected to improve NBS performance for VLCAD deficiency.

## 1. Introduction

Very-long-chain acyl-CoA dehydrogenase (VLCAD), an enzyme involved in fatty acid β-oxidation, is bound to the mitochondrial inner membrane and converts long-chain acyl-CoA supplied by carnitine palmitoyltransferase II into 2-enoyl-CoA with corresponding chain lengths. Since the first cases were reported in 1993 [1,2,3], VLCAD deficiency has been clinically classified into three phenotypes: (1) severe phenotype, characterized by cardiomyopathy associated with hypoglycemia developing in the neonatal and early infantile periods; (2) hypoglycemic phenotype, which mainly manifests during infancy and early childhood, provoking hypoketotic hypoglycemia, Reye-like encephalopathy, and, in more severe cases, cardiac arrest; and (3) myopathic phenotype, characterized by recurrent rhabdomyolysis with onset in adolescence or later [4]. The hypoglycemic phenotype can cause sudden infant death; therefore, it is included in newborn screening (NBS) panels in many countries.

Tetradecenoylcarnitine (C14:1) in dried blood specimens (DBSs) is commonly used as the primary marker for VLCAD deficiency in NBS [5,6]. Positive results are confirmed by analyzing serum or plasma acylcarnitine levels, which are usually more sensitive to detecting fatty acid oxidation disorders. Analysis of organic acid levels in urine may reveal nonketotic dicarboxylic aciduria, commonly observed during the catabolic state in patients with various fatty acid oxidation disorders. Abnormal findings in metabolite analysis are confirmed by measuring VLCAD activity or β-oxidation ability in blood mononuclear cells or skin fibroblasts and the genetic analysis of *ACADVL*. The number of *ACADVL* variants has greatly increased since introducing tandem mass spectrometry (MS/MS)-based NBS, including many novel variants of unknown clinical significance, for which quick and reliable evaluation of enzymatic function is essential [7,8,9].

NBS for VLCAD deficiency using C14:1 is associated with high false-positive rates [10]. Therefore, the ratio of C14:1 to acetylcarnitine (C2) has long been used as a secondary marker to improve specificity [1]. The utility of the C14:1 ratio to other acylcarnitines such as C16, C16-OH, C12, and C12:1 has also been reported [8,11,12,13,14,15,16]. In Japan, MS/MS-based NBS was launched as a pilot study in 1997 and introduced to the nationwide NBS program in 2013, which includes C14:1 and C14:1/C2 as markers for VLCAD deficiency [17]. We and our colleagues have offered confirmatory tests for NBS-positive infants and observed many patients with mild phenotypes that remain asymptomatic without medical management, and normal infants with false-positive test results [18,19]. In the present study, we evaluated these markers’ performance compared to various acylcarnitines and their ratios on a larger scale to identify more effective NBS markers for VLCAD deficiency.

## 2. Materials and Methods

### 2.1. Research Subjects

A total of 214 infants, who tested positive for VLCAD deficiency in NBS and were subsequently evaluated for VLCAD activity, were eligible for the present study. Among the infants showing impaired VLCAD activity, those with biallelic *ACADVL* variants were enrolled as patients with VLCAD deficiency (Group A). Infants who were heterozygous for *ACADVL* variants, including tentative cases, were enrolled as carriers (Group B). Those not confirmed by genetic analysis were excluded. Infants with normal-level VLCAD activity were enrolled as healthy infants (Group C). The utilities of levels of various acylcarnitines and free carnitine (C0) and their ratios in DBSs as NBS markers for VLCAD deficiency were evaluated by comparing the NBS data of these groups.

### 2.2. NBS Test for VLCAD Deficiency

DBSs for NBS were generally collected on postnatal day 4 or 5 according to the official protocol used since NBS for amino acid disorders began in 1977. DBSs collected using uniform filter paper (Advantec Toyo Kaisha, Tokyo, Japan) were analyzed by flow-injection electrospray-ionization MS/MS following a previously described protocol [20] with some modifications. During the pilot study conducted from 1997 to 2012, a C14:1 level of ≥0.4 nmol/mL and a C14:1/C2 ratio of ≥0.013 were used to identify newborns at risk of VLCAD deficiency. Both cutoff values corresponded to the 99.5th percentile of values in healthy newborns. Since 2013, nationwide NBS tests have been assigned to 35 regional laboratories, where the cutoff values were adjusted. The mean (±standard deviation) cutoffs used were 0.34 ± 0.06 nmol/mL and 0.013 ± 0.004 for the C14:1 level and C14:1/C2 ratio, respectively. Regarding MS/MS devices and sample preparation kits, including stable-isotope-labeled internal standards, products from several manufacturers were used in various combinations (MS/MS devices from AB Sciex, MA, USA; Waters, MA, USA; Shimadzu, Kyoto, Japan; internal standards from Siemens, Munich, Germany; PerkinElmer, MA, USA; Cambridge Isotope Laboratory, MA, USA; Sekisui Medical, Tokyo, Japan). Interlaboratory variations were evaluated regularly by external quality control tests for precision levels in MS/MS analysis, managing within ±15% of a reference value offered by the Quality Control Committee of the Japanese Society for Neonatal Screening [21].

### 2.3. Confirmatory Tests for VLCAD Deficiency

Generally, the serum acylcarnitine profile of infants with positive NBS results for VLCAD deficiency was determined by the same MS/MS method as for DBSs, using supernatant obtained after centrifugation of the mixture of serum and internal standard solution. Samples were obtained within one month after birth in most cases. In addition, the enzymatic activity of VLCAD was measured, as described in previous reports [18,22]. A high-performance liquid chromatography system (Shimadzu, Kyoto, Japan) was used to detect the production of 2-hexadecenoyl-CoA from palmitoyl-CoA and ferrocenium hexafluorophosphate (Sigma-Aldrich, Saint Louis, MO, USA), catalyzed by a crude lysate of peripheral lymphocytes isolated from NBS-positive infants. Lymphocytes were sonicated in 0.4% taurodeoxycholic acid (Sigma-Aldrich, Saint Louis MO, USA) solution to disrupt the mitochondrial membrane and liberate the enzyme. The CoA-derivatives were detected by ultraviolet spectrophotometry at 260 nm. The mean (±standard deviation) VLCAD activity in 54 normal control adults was 149.9 ± 57.1 pmol/min/10^6^ cells.

For infants with impaired VLCAD activity, further genetic analysis was performed according to the methods described in a previous report [23] with some modifications after receiving informed consent from the infants’ parents. Genomic DNA was extracted from peripheral leukocytes. All exons and flanking intron regions containing *ACADVL* were amplified using a polymerase chain reaction, and the products were analyzed by direct sequencing. In some infants, gene panel sequencing provided by a national health insurance scheme targeting fatty acid oxidation disorders [24] was performed in Kazusa DNA Research Institute (Kisarazu, Chiba, Japan) according to the preference of the pediatrician in charge.

### 2.4. Receiver Operating Characteristic Analysis

Receiver operating characteristic (ROC) curves were generated to evaluate the sensitivity and specificity of various acylcarnitines and their ratios as markers for VLCAD deficiency. ROC analysis was performed using R and RStudio statistical programs (version 4.2.2; The R Foundation, Vienna, Austria). The Youden index was used to determine the optimal cutoff value for each marker.

## 3. Results

### 3.1. Diagnosis of NBS Positivity and Levels of Currently Used Markers

The characteristics of infants enrolled in the present study with positive NBS results for VLCAD deficiency are summarized in Table 1. Detailed results of NBS and confirmatory tests for each infant are listed in Supplementary Table S1.

Group A included 95 infants with biallelic *ACADVL* variants that were categorized according to their VLCAD activity: <20% (Group A-1: N-01 to N-73), 20–40% (Group A-2: N-74 to N-92), and >40% (Group A-3: N-93 to N-95). Group B included 39 infants heterozygous for *ACADVL* variants that were categorized according to a VLCAD activity: 20–40% (Group B-1: N-96 to N-118) and >40% (Group B-2: N-119 to N-134). Group C included 41 infants with a VLCAD activity of >70% (N-135 to N-175). In this group, VLCAD activity was defined as normal without confirmation by *ACADVL* sequencing in all but one infant (N-136).

**Figure 1:**
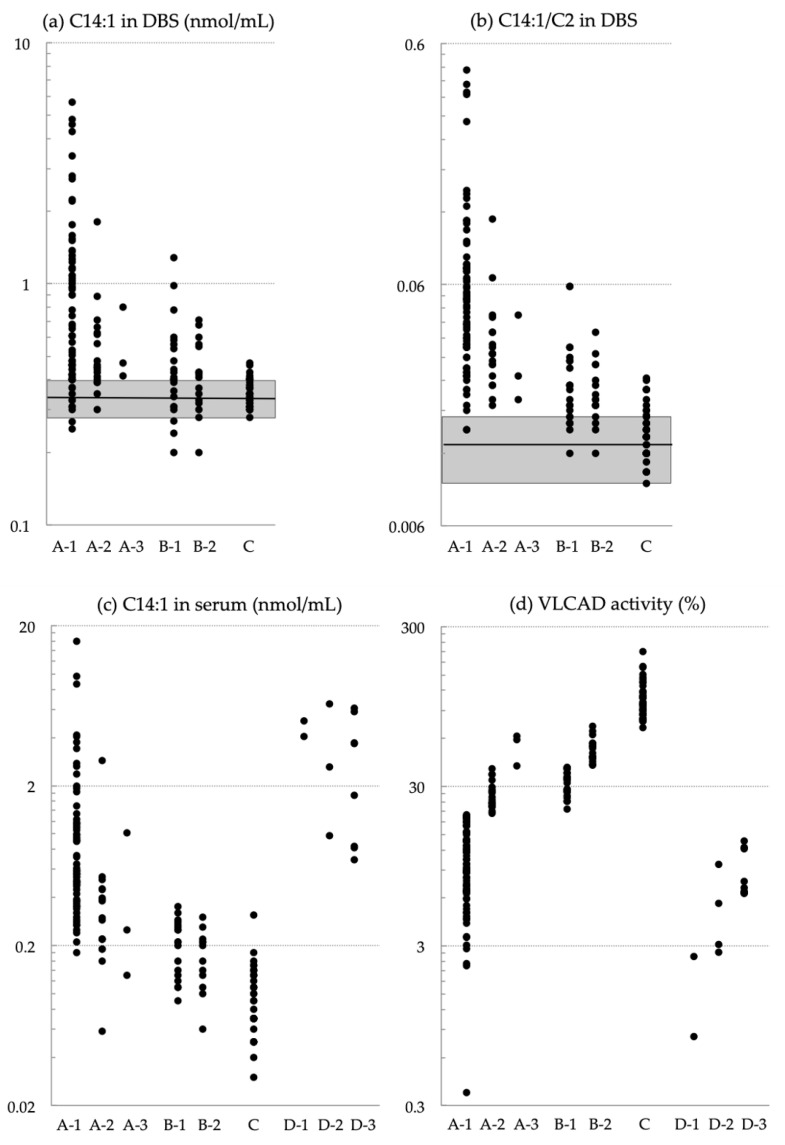
(**a**,**b**) Levels of (**a**) C14:1 and (**b**) the C14:1/C2 ratio in dried blood specimens (DBSs). (**c**,**d**) Levels of (**c**) C14:1 in serum and (**d**) VLCAD activity in lymphocytes. Groups are NBS-positive infants with biallelic *ACADVL* variants and a VLCAD activity of <20% (A-1), 20–40% (A-2), and >40% (A-3); infants heterozygous for *ACADVL* variants with a VLCAD activity of 20–40% (B-1), and >40% (B-2). Infants with a VLCAD activity of >70% without confirmation by *ACADVL* sequencing (C). Shaded squares in (**a**,**b**) indicate the following cutoff ranges (mean ± standard deviation) across 35 regional laboratories (0.34 ± 0.06 nmol/mL for C14:1 and 0.013 ± 0.004 for the C14:1/C2 ratio). Group D includes patients with VLCAD deficiency symptoms diagnosed after clinical onset. The patients were categorized by severe phenotype (D-1), hypoglycemic phenotype (D-2), and myopathic phenotype (D-3).

Figure 1 shows the levels of C14:1 and C14:1/C2 in DBSs, C14:1 levels in serum, and VLCAD activity in the study groups. None of these markers could clearly distinguish Group A from Groups B or C.

For reference, we enrolled 14 symptomatic patients as Group D (Table 1 and Figure 1), which included patients with severe phenotype (Group D-1: S-01, S-02) and those with hypoglycemic (D-2: S-03 to S-06) and myopathic phenotypes (D-3: S-07 to S-14). These patients were born before the nationwide implementation of NBS and were not enrolled in the pilot study for MS/MS-based NBS. The confirmatory test results for each symptomatic patient are listed in Supplementary Table S1.

### 3.2. Comparing the C14:1 Level and C14:1/C2 Ratio to Various Acylcarnitines in DBSs

Supplementary Table S2 shows individual acylcarnitine levels (C18:1, C18, C16, C16-OH, C14:1, C14, C12, C10, C8, C6, C4, C3, and C2) and C0 in DBSs of newborns in the present study. Supplementary Figure S1 shows scatter plots of the data. Although none of the measured parameters could clearly differentiate between infants with and without VLCAD deficiency (Group A versus C), ROC analysis revealed that the area under the ROC curve (AUC) with the optimal cutoff was highest for a decrease in C8 (0.954), followed by decreases in C10 (0.938), C6 (0.928), C2 (0.877), and an increase in C14:1 (0.881) (Table 2). These results confirmed that the currently used C14:1 level is an appropriate primary marker for VLCAD deficiency. They also suggested that medium-chain acylcarnitines may be better denominators than C2 in improving the sensitivity and specificity of C14:1.

**Figure 2:**
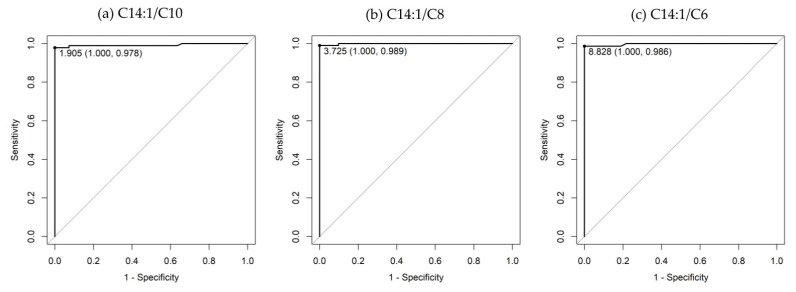
ROC curves of (**a**) C14:1/C10, (**b**) C14:1/C8, and (**c**) C14:1/C6 ratios. The area under the ROC curve for the C14:1/C10, C14:1/C8, and C14:1/C6 ratios are 0.992 (95% confidence interval, 0.9784–1), 0.999 (95% confidence interval, 0.9967–1), and 0.997 (95% confidence interval, 0.9915–1), respectively.

**Figure 3:**
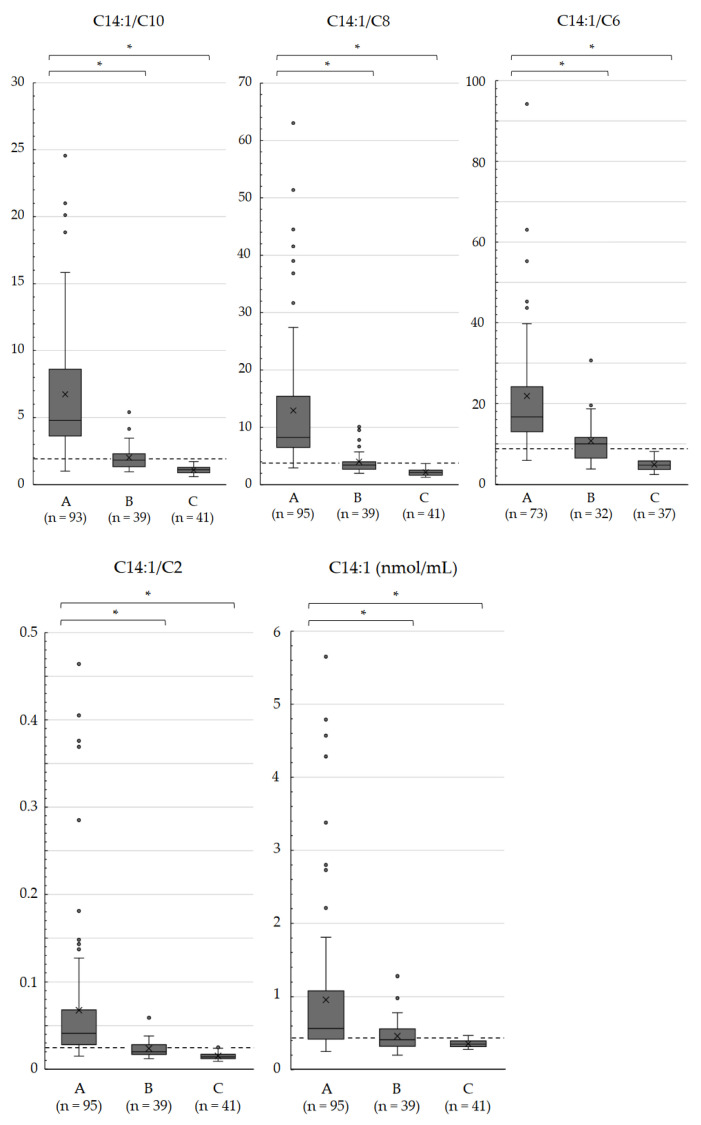
Box plots of the C14:1/C10, C14:1/C8, C14:1/C6, and C14:1/C2 ratios and C14:1 levels in dried blood specimens of newborns. Infants with biallelic *ACADVL* variants A, infants heterozygous for *ACADVL* variants B, and infants with a VLCAD activity of >70% without confirmation by *ACADVL* sequencing C. Dashed lines indicate the optimal cutoffs shown by the ROC analysis. Horizontal lines and cross marks within the boxes indicate median values and mean values, respectively. * Significant differences were indicated using Welch’s *t*-test (*p* < 0.01).

Supplementary Figure S2 shows scatter plots of C14:1 ratios to specific acylcarnitines and C0. According to the ROC analysis (Table 3), the C14:1/C8 ratio had the highest AUC (0.999), followed by the C14:1/C6 (AUC, 0.997), C14:1/C10 (AUC, 0.992), C14:1/C16-OH (AUC, 0.991), and C14:1/C2 (AUC, 0.978) ratios. Regarding sensitivity, the C14:1/C8 ratio exhibited the highest sensitivity (0.989), followed by the C14:1/C6 and C14:1/C10 ratios with sensitivities of 0.986 and 0.978, respectively. The specificities were 1.000 for all three ratios. The ROC curves of the C14:1/C10, C14:1/C8, and C14:1/C6 ratios are shown in Figure 2. The box plots comparing these ratios with the C14:1/C2 ratio and C14:1 level are shown in Figure 3.

## 4. Discussion

False-positive results in NBS can cause unnecessary anxiety for parents; therefore, markers that strike the best balance between sensitivity and specificity must be pursued. In the present study, we compared the levels and ratios of various acylcarnitines and C0 in the DBSs of 175 infants. Increased C14:1 levels exhibited the highest AUC values among long-chain acylcarnitines, which accumulate in patients with VLCAD deficiency. We also found that the AUC was higher for decreases in the medium-chain acylcarnitines C10, C8, and C6. As expected from these results, further analysis revealed that the AUCs for the C14:1/C10, C14:1/C8, and C14:1/C6 ratios were higher than those for the C14:1 level and C14:1/C2 ratio. The AUC for the C14:1/C16-OH ratio was also higher than that for the C14:1/C2 ratio. However, the C16-OH levels in newborn DBSs were too low for accurate measurements compared to other acylcarnitines included in NBS. The C14:1/C10, C14:1/C8, and C14:1/C6 ratios are promising markers, which is reasonable given the substrate specificity of VLCAD [25].

Considering the clinical symptoms of VLCAD deficiency and the availability of effective medical management, preventing false-negative results should be prioritized. Table 4 summarizes performance of the C14:1 level and the C14:1/C2, C14:1/C10, C14:1/C8, and C14:1/C6 ratios with their optimal cutoffs applied to the NBS data of infants in Groups A and C. The C14:1/C10 ratio yielded false-negative results in two patients, including one patient in Group A-2 (N-79) and another in Group A-3 (N-95). The C14:1/C8 and C14:1/C6 ratios yielded false-negative results in only one patient (N-95) who exhibited the highest VLCAD activity in Group A (62.0%). Our study cohort included infants with an indeterminate diagnosis (Group B) whose data were excluded from the ROC analysis. Our evaluation, specifically in Group B, indicated that the positivity rates based on the C14:1/C10, C14:1/C8, and C14:1/C6 ratios were 0.565, 0.478, and 0.571, respectively, in Group B-1 and 0.188, 0.250, and 0.455, respectively, in Group B-2. Assuming that patients with potential compound heterozygosity were more likely to be included in Group B-1 than in Group B-2, these ratios may also help detect this subset of patients with the lowest increase in false-positive rates.

Few studies outside Japan have recommended the C14:1 ratio to medium-chain acylcarnitines as NBS markers for VLCAD deficiency. Liebig et al. indicated that the C14:1/C8 and C14:1/C4 ratios were helpful, albeit without concrete data [7]. Boneh et al. detected six patients with VLCAD deficiency among 189,000 newborns and found that the C14:1 and C14 levels and C14:1/C10 ratio were elevated in these patients’ DBSs. However, the authors did not mention other acylcarnitines or their ratios [26]. Spiekerkoetter et al. presented data on a second-tier parameter [(C14 + C14:1 + C14:2)/(C8 + C6)] in two patients identified by NBS; however, this parameter had limited utility [8]. One study from China used the C14:1/C8 ratio as an NBS marker for VLCAD deficiency; however, details on its performance, such as its false-positive rate or evidence for choosing this marker, were not disclosed [27].

One major limitation of our study was the lack of reference values for NBS markers in VLCAD deficiency, except those for the currently used C14:1 level and C14:1/C2 ratio. The C14:1/C10, C14:1/C8, and C14:1/C6 ratio performances must be evaluated using nationwide NBS data. In addition, DBS sampling is conducted on postnatal day 4 or 5 in Japan, while it is scheduled between 24 and 48 h after birth in many countries [28] because the accumulation of fatty acylcarnitines in newborn blood rapidly decreases after feeding initiation. Therefore, our findings should be further validated according to specific countries’ NBS protocols.

## 5. Conclusions

In Japan, NBS for VLCAD deficiency, which was initially launched as a pilot study in 1997, has become a nationwide healthcare service since 2013. We confirmed VLCAD deficiency in 95 out of 175 infants based on NBS. The currently used C14:1 level and C14:1/C2 ratio markers are associated with high false-positive rates. Therefore, we performed an extensive evaluation to compare acylcarnitine levels of various chain lengths, ranging from C2 to C18, and C0. We also compared ratios of C14:1 to other acylcarnitines and C0 in the DBSs of 175 infants. Our analyses revealed that the C14:1/C10, C14:1C8, and C14:1/C6 ratios were most effective based on lower false-positive rates. Our future plans include retrospective and prospective nationwide studies to evaluate their performance. We ultimately aim to set appropriate cutoffs and reduce false-positive rates for VLCAD deficiency in Japan based on NBS results.

## Figures and Tables

**Table 1:** Comparison between NBS-positive infants and symptomatic patients according to the classifications used in the present study.

Groups and Descriptions of Infants with NBS-Positive Results for VLCAD Deficiency (Groups A–C) and Symptomatic Patients (Group D)		*n*	Case ID *^1^	VLCAD Activity *^2^
Group A-1	Biallelic *ACADVL* variants detected, VLCAD activity lower than 20%	73	N-01 to N-73	0.36–19.88% (10.28 ± 5.13)
Group A-2	Biallelic *ACADVL* variants detected, VLCAD activity between 20% and 40%	19	N-74 to N-92	20.32–38.70% (27.01 ± 5.35)
Group A-3	Biallelic *ACADVL* variants detected, VLCAD activity higher than 40%	3	N-93 to N-95	40.55–58.73%, 62.00%
Group B-1	Heterozygous *ACADVL* variants detected, VLCAD activity between 20% and 40%	23	N-96 to N-118	21.62–39.35% (31.80 ± 5.56)
Group B-2	Heterozygous *ACADVL* variants detected, VLCAD activity higher than 40%	16	N-119 to N-134	40.66–71.32% (51.74 ± 9.17)
Group C	VLCAD activity higher than 70% without confirmation with *ACADVL* sequencing	41	N-135 to N-175	70.27–208.67% (107.96 ± 29.43)
Group D-1	Severe phenotype	2	S-01, S-02	0.81–2.59%
Group D-2	Hypoglycemic phenotype	4	S-03 to S-06	2.75–9.75%
Group D-3	Myopathic phenotype	8	S-07 to S-14	6.41–13.72%

Abbreviations: NBS, newborn screening; VLCAD, very-long-chain acyl-CoA dehydrogenase. *^1^ Detailed data on NBS and confirmatory tests for each infant are presented in Supplementary Tables S1 and S2. Diagnostic findings of symptomatic patients are presented in Supplementary Table S1. *^2^ Mean (± standard deviation) VLCAD activity in 54 normal control adults is 149.9 ± 57.1 pmol/min/10^6^ cells.

**Table 2:** ROC analysis of specific acylcarnitine levels and free carnitine in dried blood specimens of newborns.

Characteristic	Acylcarnitine and Free Carnitine Levels													
	C18:1	C18	C16	C16-OH	C14:1	C14	C12	C10	C8	C6	C4	C3	C2	C0
Optimal cutoff (nmol/mL)	1.358	0.853	2.627	0.031	0.417	0.421	0.403	0.215	0.110	0.047	0.175	1.230	21.859	17.780
Sensitivity	0.435	0.624	0.543	0.840	0.768	0.419	0.329	0.892	0.853	0.808	0.686	0.613	0.863	0.696
Specificity	0.795	0.634	0.902	0.732	0.927	0.951	0.927	0.976	0.976	0.973	0.919	0.512	0.756	0.732
Area under the ROC curve	0.594	0.626	0.744	0.807	0.881	0.681	0.432	0.938	0.954	0.928	0.840	0.518	0.877	0.749

Abbreviations: ROC, receiver operating characteristic; C0, free carnitine.

**Table 3:** ROC analysis of C14:1 ratios to specific acylcarnitines and C0 in dried blood specimens of newborns.

Characteristic	Acylcarnitine Ratios												
	C14:1/ C18:1	C14:1/ C18	C14:1/ C16	C14:1/ C16-OH	C14:1/ C14	C14:1/ C12	C14:1/ C10	C14:1/ C8	C14:1/ C6	C14:1/ C4	C14:1/ C3	C14:1/ C2	C14:1/ C0
Optimal cutoff	0.319	0.600	0.153	19.444	1.398	1.264	1.905	3.725	8.828	1.959	0.394	0.023	0.025
Sensitivity	0.788	0.634	0.915	0.926	0.709	0.941	0.978	0.989	0.986	0.914	0.559	0.895	0.696
Specificity	0.949	0.854	0.976	1.000	0.805	0.902	1.000	1.000	1.000	0.892	0.951	0.951	0.610
Area under the ROC curve	0.919	0.797	0.972	0.991	0.814	0.972	0.992	0.999	0.997	0.942	0.768	0.978	0.694

**Table 4:** Application of the C14:1 level and C14:1/C2, C14:1/C10, C14:1/C8, and C14:1/C6 ratios to NBS data using optimal cutoffs determined by ROC analysis.

Index: C14:1 ≥ 0.417 nmol/mL				
**Group A**	Data Available 95	Positive 73	Negative 22	Sensitivity 0.768
**Group C**	Data Available 41	Positive 3	Negative 38	Specificity 0.927
**Group A + C**	Positive predictive value 0.961		Negative predictive value 0.633	
**Group** **B-1/B-2**	Data Available 23/16	Positive 10/8	Negative 13/8	Positive rate 0.435/0.500
**Index: C14:1/C2 ≥ 0.023**				
**Group A**	Data Available 95	Positive 85	Negative 10	Sensitivity 0.895
**Group C**	Data Available 41	Positive 2	Negative 39	Specificity 0.951
**Group A + C**	Positive predictive value 0.977		Negative predictive value 0.796	
**Group** **B-1/B-2**	Data Available 23/16	Positive 11/6	Negative 12/10	Positive rate 0.478/0.375
**Index: C14:1/C10 ≥ 1.905**				
**Group A**	Data Available 93	Positive 91	Negative 2	Sensitivity 0.978
**Group C**	Data Available 41	Positive 0	Negative 41	Specificity 1
**Group A + C**	Positive predictive value 1		Negative predictive value 0.953	
**Group** **B-1/B-2**	Data Available 23/16	Positive 13/3	Negative 10/13	Positive rate 0.565/0.188
**Index: C14:1/C8 ≥ 3.725**				
**Group A**	Data Available 95	Positive 94	Negative 1	Sensitivity 0.989
**Group C**	Data Available 41	Positive 0	Negative 41	Specificity 1
**Group A + C**	Positive predictive value 1		Negative predictive value 0.976	
**Group** **B-1/B-2**	Data Available 23/16	Positive 11/4	Negative 12/12	Positive rate 0.478/0.250
**Index: C14:1/C6 ≥ 8.828**				
**Group A**	Data Available 73	Positive 72	Negative 1	Sensitivity 0.986
**Group C**	Data Available 37	Positive 0	Negative 37	Specificity 1
**Group A + C**	Positive predictive value 1		Negative predictive value 0.974	
**Group** **B-1/B-2**	Data Available 21/11	Positive 12/5	Negative 9/6	Positive rate 0.571/0.455

## Data Availability

The data presented in this study are available upon request from the corresponding author.

## Supplementary Materials

The following supporting information can be downloaded at: https://www.mdpi.com/article/10.3390/ijns10010015/s1. Table S1: Results of newborn screening and confirmatory tests of the infants enrolled in the present study and the diagnostic data of symptomatic patients acquired after the clinical onset used as reference; Table S2: Concentrations of acylcarnitines and free carnitine in dried blood specimens of newborns; Figure S1: Levels of various acylcarnitines and free carnitine in dried blood specimens of newborns; Figure S2: Ratios of C14:1 to various acylcarnitines and free carnitine in dried blood specimens of newborns.
