# Supplementary material for: Using the C14:1/Medium-Chain Acylcarnitine Ratio Instead of C14:1 to Reduce False-Positive Results for Very-Long-Chain Acyl-CoA Dehydrogenase Deficiency in Newborn Screening in Japan
Source: Int J Neonatal Screen. 2024 Feb 20;10(1):15. doi: 10.3390/ijns10010015 (PMC10885094; doi:10.3390/ijns10010015)
Supplement: Supplementary file 1 [file IJNS-10-00015-s001.zip › FigureS1.pdf]

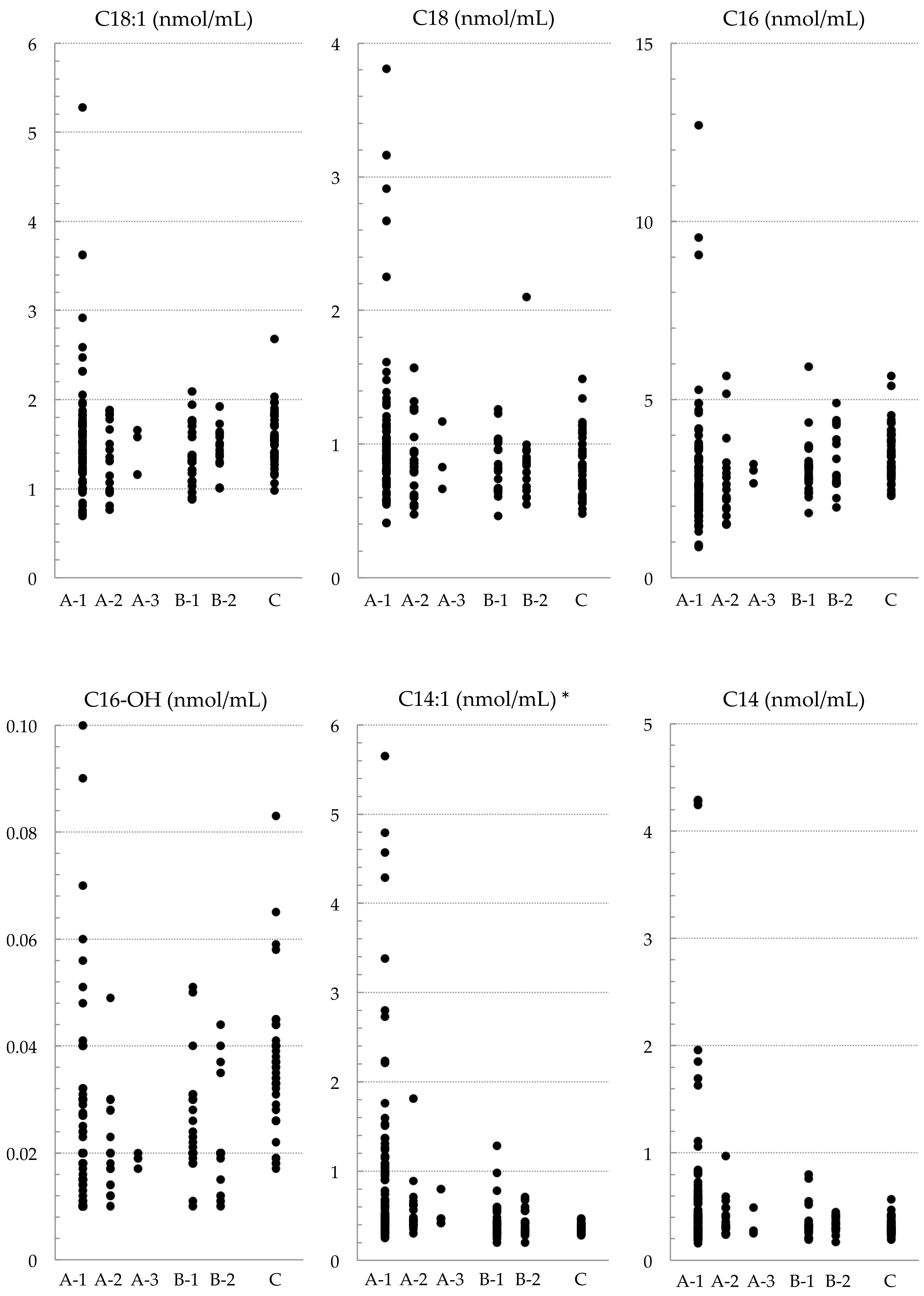

C18:1 (nmol/mL)
0
1
2
3
4
5
6
A-1 A-2 A-3 B-1 B-2 C
C18 (nmol/mL)
0
1
2
3
4
A-1 A-2 A-3 B-1 B-2 C
C16 (nmol/mL)
0
5
10
15
A-1 A-2 A-3 B-1 B-2 C
C16-OH (nmol/mL)
0
0.02
0.04
0.06
0.08
0.10
A-1 A-2 A-3 B-1 B-2 C
C14:1 (nmol/mL) *
0
1
2
3
4
5
6
A-1 A-2 A-3 B-1 B-2 C
C14 (nmol/mL)
0
1
2
3
4
5
A-1 A-2 A-3 B-1 B-2 C

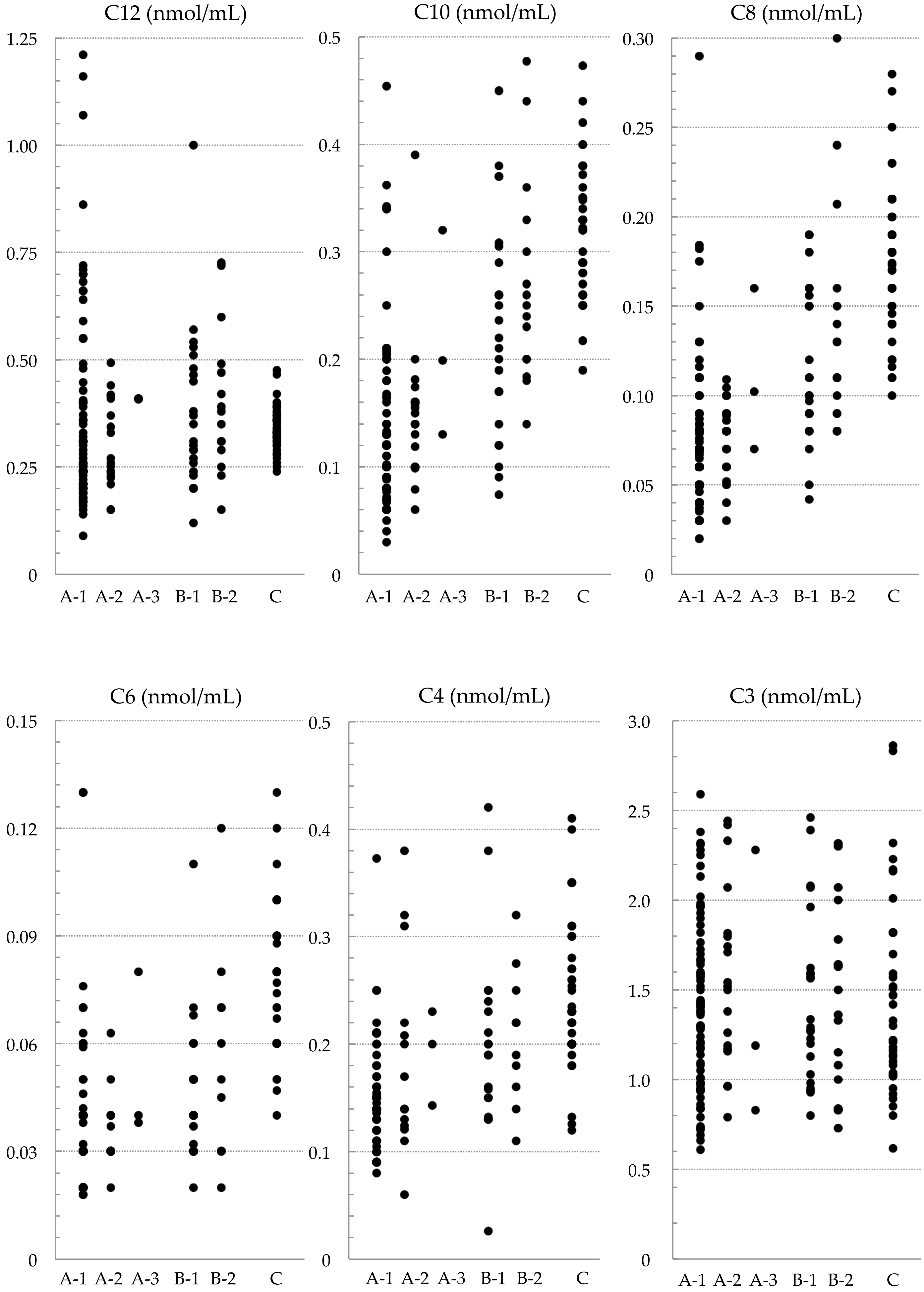
C12 (nmol/mL)
0
0.25
0.50
0.75
1.00
1.25
C10 (nmol/mL)
0
0.1
0.2
0.3
0.4
0.5
C8 (nmol/mL)
0
0.05
0.10
0.15
0.20
0.25
0.30
C6 (nmol/mL)
0
0.03
0.06
0.09
0.12
0.15
C4 (nmol/mL)
0
0.1
0.2
0.3
0.4
0.5
C3 (nmol/mL)
0
0.5
1.0
1.5
2.0
2.5
3.0
A-1 A-2 A-3 B-1 B-2 C

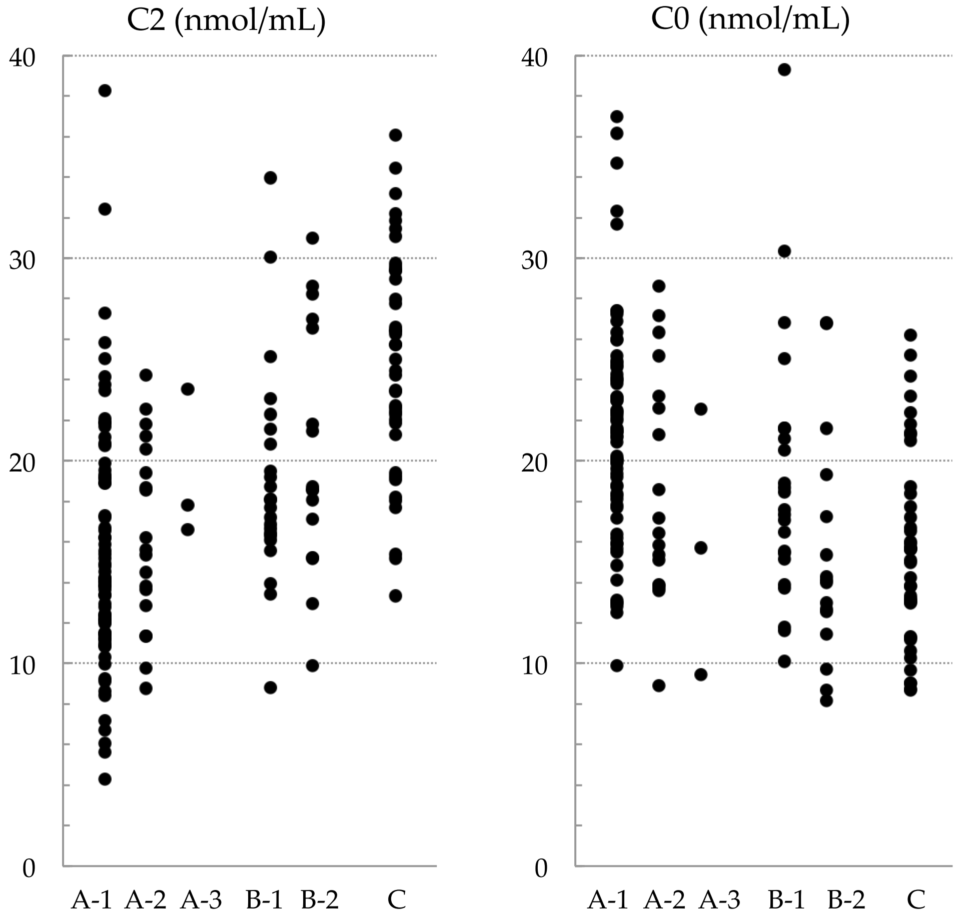

Figure S1. Levels of various acylcarnitines and free carnitine in dried blood specimens of newborns. Infants with biallelic *ACADVL* variants and a VLCAD activity of <20% (A-1), 20%–40% (A-2), and >40% (A-3); infants heterozygous for *ACADVL* variants with a VLCAD activity of 20%–40% (B-1) and >40% (B-2); infants with a VLCAD activity of >70% without confirmation by *ACADVL* sequencing (C)

C0, free carnitine

* Levels of C14:1 are also shown on a logarithmic scale in Figure 1a.
