# Supplementary material for: Using the C14:1/Medium-Chain Acylcarnitine Ratio Instead of C14:1 to Reduce False-Positive Results for Very-Long-Chain Acyl-CoA Dehydrogenase Deficiency in Newborn Screening in Japan
Source: Int J Neonatal Screen. 2024 Feb 20;10(1):15. doi: 10.3390/ijns10010015 (PMC10885094; doi:10.3390/ijns10010015)
Supplement: Supplementary file 1 [file IJNS-10-00015-s001.zip › FigureS2.pdf]

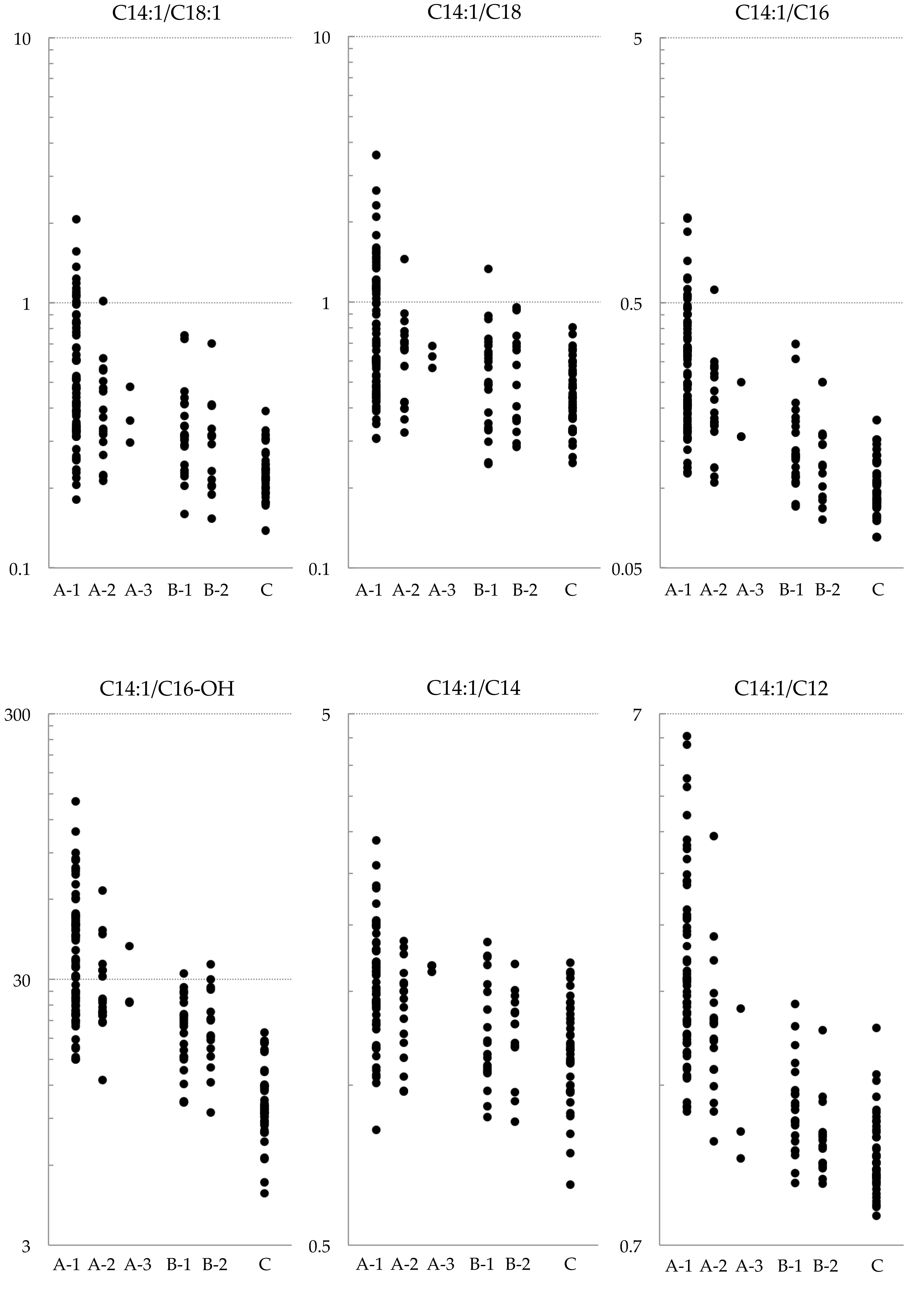
C14:1/C18:1
10
1
0.1
A-1 A-2 A-3 B-1 B-2 C
C14:1/C18
10
1
0.1
A-1 A-2 A-3 B-1 B-2 C
C14:1/C16
5
0.5
0.05
A-1 A-2 A-3 B-1 B-2 C
C14:1/C16-OH
300
30
3
A-1 A-2 A-3 B-1 B-2 C
C14:1/C14
5
0.5
A-1 A-2 A-3 B-1 B-2 C
C14:1/C12
7
0.7
A-1 A-2 A-3 B-1 B-2 C

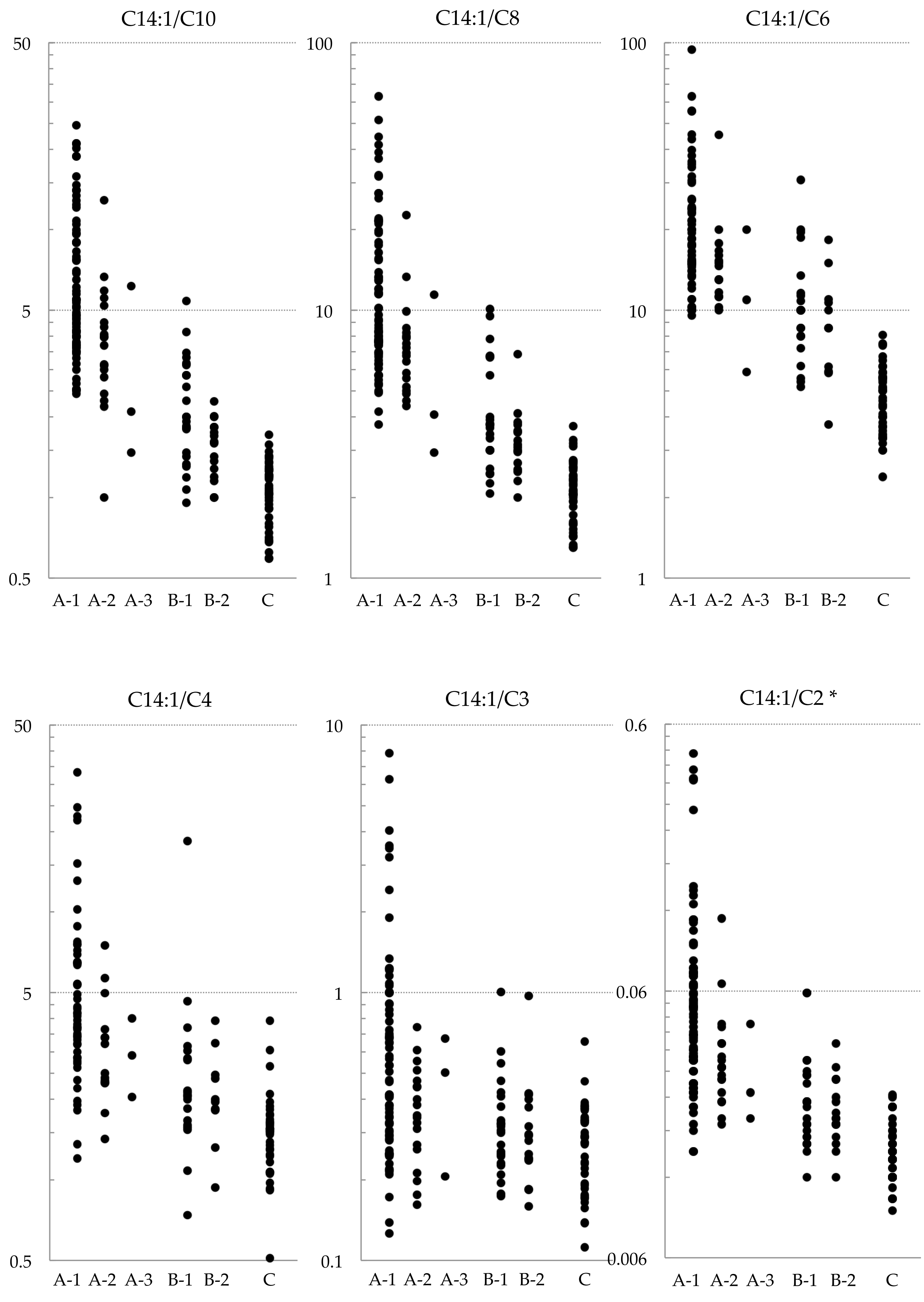
C14:1/C10
50
5
0.5
A-1 A-2 A-3 B-1 B-2 C
C14:1/C8
100
10
1
A-1 A-2 A-3 B-1 B-2 C
C14:1/C6
100
10
1
A-1 A-2 A-3 B-1 B-2 C
C14:1/C4
50
5
0.5
A-1 A-2 A-3 B-1 B-2 C
C14:1/C3
10
1
0.1
A-1 A-2 A-3 B-1 B-2 C
C14:1/C2 *
0.6
0.06
0.006
A-1 A-2 A-3 B-1 B-2 C

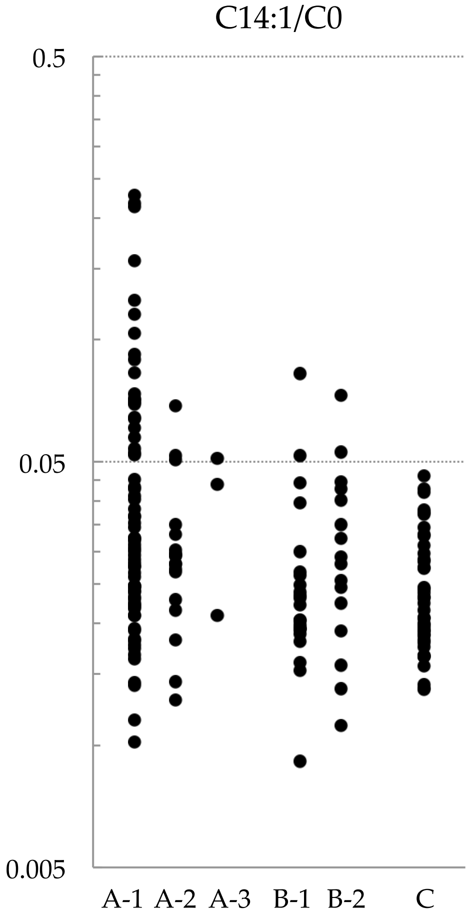

Figure S2. Ratios of C14:1 to various acylcarnitines and free carnitine in dried blood specimens of newborns. Infants with biallelic *ACADVL* variants and a VLCAD activity of <20% (A-1), 20%–40% (A-2), and >40% (A-3); infants heterozygous for *ACADVL* variants with a VLCAD activity of 20%–40% (B-1) and >40% (B-2); infants with a VLCAD activity of >70% without confirmation by *ACADVL* sequencing (C)

C0, free carnitine

* The graph of the C14:1/C2 ratio is also shown in Figure 1b.
