# Supplementary material for: Using the C14:1/Medium-Chain Acylcarnitine Ratio Instead of C14:1 to Reduce False-Positive Results for Very-Long-Chain Acyl-CoA Dehydrogenase Deficiency in Newborn Screening in Japan
Source: Int J Neonatal Screen. 2024 Feb 20;10(1):15. doi: 10.3390/ijns10010015 (PMC10885094; doi:10.3390/ijns10010015)
Supplement: Supplementary file 1 [file IJNS-10-00015-s001.zip › TableS1.pdf]

Table S1. Results of newborn screening and confirmatory tests of the infants enrolled in the present study and the diagnostic data of symptomatic patients acquired after the clinical onset used as reference.

Case ID	Current NBS Indices and Cutoff (Mean ± SD in 35 Laboratories)		Serum C14:1 (nmol/mL)	VLCAD Activity (%) [1]	*ACADVL* Genotype [2]				Diagnoses of NBS-Positive Infants
					Variant 1		Variant 2		
	C14:1 (nmol/mL) 0.34 ± 0.06	C14:1/C2 0.013 ± 0.004			cDNA	Amino acid	cDNA	Amino acid	
NBS-positive infants									
N-01	3.38	0.285	9.67	0.36	878+1G > C		919A > G	T307A	Patient
N-02	1.26	0.137	3.76	2.26	865G > A	G289R	1820G>C	C607S	Patient
N-03	4.29	0.376	15.93	2.32	996dupT	A333Cfs	1358G > A	R453Q	Patient
N-04	0.78	0.063	0.90	2.87	790A > G	K264E	996dupT	A333Cfs	Patient
N-05	5.65	0.405	8.66	3.04	996dupT	A333Cfs	1793A > G	H598R	Patient
N-06	0.96	0.044	0.98	3.41	1246G > A	A416T	1246G > A	A416T	Patient
N-07	0.65	0.049	ND	3.45	623-3C > A		865G > A	G289R	Patient
N-08	0.42	0.022	0.50	4.15	1793A > G	H598R	1808G > C	C603S	Patient
N-09	2.80	0.464	4.08	4.40	1349G > A	R450H	1349G > A	R450H	Patient
N-10	2.23	0.089	1.11	4.50	996dupT	A333Cfs	1246G > A	A416T	Patient
N-11	2.73	0.143	0.94	4.62	787G > A	A263T	1427G > A	G476D	Patient
N-12	0.40	0.033	0.27	4.84	334T > C	F112L	520G > A<br>1434G > A	V174M<br>M478I	Patient
N-13	0.40	0.024	2.75	4.87	790A > G	K264E	1349G > A	R450H	Patient
N-14	4.79	0.148	0.99	5.14	708_709delCT	C237Wfs	1246G > A	A416T	Patient
N-15	0.61	0.051	1.84	5.38	538G > A	A180T	1002G > A	M334I	Patient
N-16	0.90	0.070	1.49	5.93	1077G > A	A359=	1077G > A	A359=	Patient
N-17	0.46	0.041	1.17	5.94	538G > A	A180T	1246G > A	A416T	Patient
N-18	2.21	0.111	2.36	6.38	865G > A	G289R	1732_1734delATG	M578del	Patient
N-19	0.42	0.022	0.65	6.56	746G > T	W249L	1349G > A	R450H	Patient
N-20	1.08	0.108	1.93	6.59	1246G > A	A416T	1246G > A	A416T	Patient
N-21	1.17	0.073	1.15	6.61	1246G > A	A416T	1793A > G	H598R	Patient
N-22	0.31	0.073	0.91	6.63	890AGA[2]	K299del	1820G > C	C607S	Patient
N-23	0.68	0.026	0.54	6.75	852G > A	V284=	1434G > A	M478I	Patient
N-24	1.37	0.070	0.34	6.91	538G > A	A180T	790A > G	K264E	Patient
N-25	0.33	0.030	0.49	7.15	709T > C	C237R	709T > C	C237R	Patient
N-26	1.16	0.056	0.61	7.20	790A > G	K264E	996dupT	A333Cfs	Patient
N-27	0.37	0.025	0.45	7.42	548G > A	S183N	779C > T	T260M	Patient
N-28	1.31	0.127	2.66	7.71	553G > A	G185S	709T > C	C237R	Patient
N-29	0.61	0.054	0.92	8.01	266 C > A	P89Q	865 G > A	G289R	Patient
N-30	0.95	0.070	1.33	8.10	1246G > A	A416T	1532G > A	R511Q	Patient
N-31	0.97	0.059	1.99	8.63	1246G > A	A416T	1349G > A	R450H	Patient
N-32	1.08	0.062	0.72	8.72	334T > C	F112L	503G > A	G168D	Patient
N-33	0.46	0.055	4.17	8.73	370delG	A124Lfs	709T > C	C237R	Patient
N-34	1.37	0.036	0.38	8.87	1246G > A	A416T	1820G > C	C607S	Patient
N-35	0.52	0.037	0.30	8.98	637G > A	A213T	1820G > C	C607S	Patient
N-36	0.48	0.034	0.50	9.19	652G > A	E218K	865G > A	G289R	Patient
N-37	0.57	0.030	0.18	9.36	1246G > A	A416T	1434G > A	M478I	Patient
N-38	1.03	0.068	0.73	9.75	553G > A	G185S	1820G > C	C607S	Patient
N-39	0.67	0.041	0.42	9.87	727C > T	L243F	1820G > C	C607S	Patient
N-40	1.24	0.101	1.06	10.46	842C > G	A281G	1246G > A	A416T	Patient
N-41	0.25	0.034	0.39	10.46	307A > G	K103E	854A > G	E285G	Patient
N-42	0.40	0.015	0.51	11.04	996dupT	A333Cfs	1434G > A	M478I	Patient
N-43	0.44	0.078	0.50	11.56	614T > G	L205R	1820G > C	C607S	Patient
N-44	0.48	0.035	1.23	11.60	709T > C	C237R	1246G > A	A416T	Patient
N-45	4.57	0.369	3.42	12.01	779C > T	T260M	996dupT	A333Cfs	Patient
N-46	0.27	0.025	0.54	12.09	1357C > T	R453*	1434G > A	M478I	Patient
N-47	0.48	0.042	0.95	12.31	1434G > A	M478I	1532G > A	R511Q	Patient
N-48	1.15	0.053	ND	12.65	1144A > C	K382Q	1820G > C	C607S	Patient
N-49	1.76	0.091	0.24	12.78	709T > C	C237R	1532G > A	R511Q	Patient

N-50	0.46	0.021	0.25	13.47	548G > A	S183N	1246G > A	A416T	Patient
N-51	0.90	0.033	ND	13.61	865G > A	G289R	1820G > C	C607S	Patient
N-52	0.46	0.033	1.09	13.63	1820G > C	C607S	1793A > G	H598R	Patient
N-53	0.41	0.027	0.27	13.65	745T > C	W249R	1820G > C	C607S	Patient
N-54	0.37	0.025	0.38	13.65	854A > G	E285G	1820G > C	C607S	Patient
N-55	1.05	0.064	ND	13.85	1078-2A > G		1700G > A	R567Q	Patient
N-56	0.35	0.052	0.21	13.96	334T > C	F112L	1678+3_1678+6del AAGT		Patient
N-57	0.74	0.052	0.59	13.98	1144A > C	K382Q	1434G > A	M478I	Patient
N-58	0.61	0.071	0.35	14.90	996dupT	A333Cfs	1820G > C	C607S	Patient
N-59	0.33	0.018	0.59	15.21	278-31_278-18del GCCTGACCAGCCTG		1434G > A	M478I	Patient
N-60	0.46	0.019	0.35	15.55	506T > C	M169T	609delC	K204Sfs	Patient
N-61	0.42	0.027	0.29	15.66	538G > A	A180T	1820G > C	C607S	Patient
N-62	1.59	0.058	0.28	16.93	1077G > A	A359=	1820G > C	C607S	Patient
N-63	0.53	0.046	0.36	17.02	996dupT	A333Cfs	1820G > C	C607S	Patient
N-64	0.42	0.039	0.48	17.17	709T > C	C237R	1246G > A	A416T	Patient
N-65	1.53	0.111	0.32	17.81	334T > C	F112L	334T > C	F112L	Patient
N-66	1.51	0.068	1.17	17.91	1269G > A	S423=	1820G > C	C607S	Patient
N-67	0.31	0.034	0.56	18.15	1349G > A	R450H	1820G > C	C607S	Patient
N-68	1.00	0.048	0.47	18.70	507_527delGCATGACCT TGGCGTGGGCAT	M169_ G175del	1226C > T	T409M	Patient
N-69	0.30	0.026	0.48	18.93	709T > C	C237R	709T > C	C237R	Patient
N-70	0.44	0.036	0.56	19.28	71_77delTCACGGC	L24Rfs	1434G > A	M478I	Patient
N-71	0.51	0.040	0.58	19.42	1246G > A	A416T	1434G > A	M478I	Patient
N-72	0.40	0.027	0.37	19.74	538G > A	A180T	1820G > C	C607S	Patient
N-73	0.40	0.015	0.56	19.88	538G > A	A180T	1820G > C	C607S	Patient
N-74	0.43	0.044	0.45	20.32	1358G > A	R453Q	1820G > C	C607S	Patient
N-75	1.81	0.112	2.86	21.00	779C > T	T260M	1246G > A	A416T	Patient
N-76	0.35	0.034	0.54	22.32	1349G > A	R450H	1820G > C	C607S	Patient
N-77	0.46	0.025	0.45	22.57	329C > T	S110F	1434G > A	M478I	Patient
N-78	0.30	0.023	0.16	22.66	865G > A	G289R	1246G > A	A416T	Patient
N-79	0.39	0.045	0.22	23.56	1144A > C	K382Q	1820G > C	C607S	Patient
N-80	0.41	0.019	0.22	23.70	277+1G > A		1226C > T	T409M	Patient
N-81	0.45	0.031	0.19	23.75	334T > C	F112L	1820G > C	C607S	Patient
N-82	0.40	0.029	0.52	24.76	854A > G	E285G	1820G > C	C607S	Patient
N-83	0.39	0.020	0.40	25.65	1246G > A	A416T	1820G > C	C607S	Patient
N-84	0.48	0.031	0.84	26.41	796C > G<br>1153C > T	P266A<br>R385W	1820G > C	C607S	Patient
N-85	0.45	0.033	ND	27.30	1434G > A	M478I	1820G > C	C607S	Patient
N-86	0.63	0.028	0.22	27.90	1246G > A	A416T	1820G > C	C607S	Patient
N-87	0.44	0.038	0.39	28.62	1226C > T	T409M	1748C > T	S583L	Patient
N-88	0.62	0.028	0.19	29.77	1434G > A	M478I	1820G > C	C607S	Patient
N-89	0.71	0.038	0.06	32.77	334T > C	F112L	1349G > A	R450H	Patient
N-90	0.89	0.064	0.38	35.60	1226C > T	T409M	1748C > T	S583L	Patient
N-91	0.66	0.031	0.29	35.81	996dupT	A333Cfs	1226C > T	T409M	Patient
N-92	0.56	0.023	0.30	38.70	1226C > T	T409M	1793A > G	H598R	Patient
N-93	0.42	0.025	0.25	40.55	1405C > T	R469W	1332+10G > A		Patient
N-94	0.80	0.045	1.02	58.73	334T > C	F112L	1009C > T	L337F	Patient
N-95	0.47	0.020	0.13	62.00	746G > T	W249L	1592G > A	R531Q	Patient
N-96	0.41	0.020	0.27	21.62	1349G > A	R450H	No variant		Carrier
N-97	0.30	0.019	0.13	24.17	422C > A	A141D	No variant		Carrier
N-98	0.60	0.027	ND	24.30	1097G > C	R366P	No variant		Carrier
N-99	0.59	0.017	0.21	25.41	305T > C	L102P	No variant		Carrier
N-100	0.24	0.017	0.20	26.35	1078-2A > G		No variant		Carrier
N-101	0.20	0.023	ND	27.69	1820G > C	C607S	No variant		Carrier
N-102	0.40	0.023	0.28	27.92	842C > G	A281G	No variant		Carrier
N-103	0.36	0.012	0.25	28.20	996dupT	A333Cfs	No variant		Carrier

N-104	0.48	0.029	0.26	28.67	722A > G	Y241C	No variant	Carrier
N-105	0.98	0.059	ND	28.71	1226C > T	T409M	No variant	Carrier
N-106	1.28	0.059	ND	31.68	1096C > T	R366C	No variant	Carrier
N-107	0.39	0.016	0.25	32.76	1078-2A > G		No variant	Carrier
N-108	0.43	0.023	0.16	33.33	538G > A	A180T	No variant	Carrier
N-109	0.31	0.016	0.21	33.82	1226C > T	T409M	No variant	Carrier
N-110	0.27	0.015	0.14	33.99	1001T > C	M334T	No variant	Carrier
N-111	0.44	0.033	ND	34.65	1246G > A	A416T	No variant	Carrier
N-112	0.34	0.018	0.32	36.40	609delC	K204Sfs	No variant	Carrier
N-113	0.40	0.019	0.35	36.42	1322_1323delinsTA	G441V	No variant	Carrier
N-114	0.56	0.033	0.12	38.81	890AGA[2]	K299del	No variant	Carrier
N-115	0.78	0.029	0.29	38.85	553G > A	G185S	No variant	Carrier
N-116	0.31	0.016	0.11	39.17	709T > C	C237R	No variant	Carrier
N-117	0.40	0.022	0.11	39.18	790A > G	K264E	No variant	Carrier
N-118	0.54	0.030	0.09	39.35	1820G > C	C607S	No variant	Carrier
N-119	0.60	0.019	ND	40.66	1246G > A	A416T	No variant	Carrier
N-120	0.42	0.023	0.26	41.32	1106T > A	F369Y	No variant	Carrier
N-121	0.43	0.028	0.30	42.72	553G > A	G185S	No variant	Carrier
N-122	0.71	0.038	0.11	44.50	1434G > A	M478I	No variant	Carrier
N-123	0.68	0.028	ND	45.59	1349G > A	R450H	No variant	Carrier
N-124	0.41	0.019	ND	45.95	136C > T	Q46*	No variant	Carrier
N-125	0.33	0.012	0.14	46.04	790A > G	K264E	No variant	Carrier
N-126	0.32	0.024	0.20	48.33	1375C > T	R459W	No variant	Carrier
N-127	0.35	0.017	0.06	52.29	865G > A	G289R	No variant	Carrier
N-128	0.30	0.02	0.21	53.64	1144A > C	K382Q	No variant	Carrier
N-129	0.28	0.015	0.10	53.73	298_299delCA	Q100Vfs	No variant	Carrier
N-130	0.20	0.020	0.16	55.86	334T > C	F112L	No variant	Carrier
N-131	0.56	0.031	0.22	56.08	71_77delTCACGGC	L24Rfs	No variant	Carrier
N-132	0.55	0.019	ND	63.29	890AGA[2]	K299del	No variant	Carrier
N-133	0.43	0.016	0.13	66.59	1820G > C	C607S	No variant	Carrier
N-134	0.37	0.021	0.21	71.32	1820G > C	C607S	No variant	Carrier
N-135	0.28	0.009	ND	70.27		Not analyzed		Normal
N-136	0.40	0.022	0.07	76.82	No variant		No variant	Normal
N-137	0.38	0.014	ND	77.21		Not analyzed		Normal
N-138	0.35	0.010	0.05	79.08		Not analyzed		Normal
N-139	0.43	0.014	0.06	79.14		Not analyzed		Normal
N-140	0.41	0.017	ND	79.26		Not analyzed		Normal
N-141	0.42	0.018	ND	80.43		Not analyzed		Normal
N-142	0.34	0.012	0.31	84.09		Not analyzed		Normal
N-143	0.30	0.022	0.15	84.72		Not analyzed		Normal
N-144	0.46	0.024	0.05	85.23		Not analyzed		Normal
N-145	0.30	0.016	0.12	85.99		Not analyzed		Normal
N-146	0.32	0.017	0.09	89.60		Not analyzed		Normal
N-147	0.47	0.022	0.07	90.34		Not analyzed		Normal
N-148	0.30	0.012	0.11	90.35		Not analyzed		Normal
N-149	0.33	0.015	0.10	90.76		Not analyzed		Normal
N-150	0.33	0.015	0.13	93.07		Not analyzed		Normal
N-151	0.32	0.013	0.07	96.59		Not analyzed		Normal
N-152	0.31	0.012	0.16	97.74		Not analyzed		Normal
N-153	0.35	0.012	0.10	98.09		Not analyzed		Normal
N-154	0.31	0.014	0.03	98.33		Not analyzed		Normal
N-155	0.40	0.018	0.07	100.68		Not analyzed		Normal
N-156	0.41	0.014	0.11	101.06		Not analyzed		Normal
N-157	0.37	0.025	ND	107.60		Not analyzed		Normal
N-158	0.35	0.014	0.07	108.30		Not analyzed		Normal

N-159	0.40	0.015	0.15	108.79	Not analyzed				Normal
N-160	0.31	0.010	0.08	108.94	Not analyzed				Normal
N-161	0.32	0.012	0.14	109.81	Not analyzed				Normal
N-162	0.30	0.016	0.04	110.44	Not analyzed				Normal
N-163	0.34	0.015	0.14	110.94	Not analyzed				Normal
N-164	0.31	0.020	0.18	115.52	Not analyzed				Normal
N-165	0.38	0.014	0.07	117.77	Not analyzed				Normal
N-166	0.34	0.019	ND	118.42	Not analyzed				Normal
N-167	0.40	0.015	ND	128.59	Not analyzed				Normal
N-168	0.33	0.012	0.11	135.15	Not analyzed				Normal
N-169	0.37	0.012	0.07	135.23	Not analyzed				Normal
N-170	0.37	0.012	0.09	140.45	Not analyzed				Normal
N-171	0.35	0.010	0.13	145.44	Not analyzed				Normal
N-172	0.37	0.016	0.05	150.32	Not analyzed				Normal
N-173	0.39	0.012	0.14	167.59	Not analyzed				Normal
N-174	0.31	0.013	0.05	169.66	Not analyzed				Normal
N-175	0.35	0.011	0.13	208.67	Not analyzed				Normal
Patients with VLCAD deficiency diagnosed after presenting with clinical symptoms									Clinical phenotype
S-01	Not enrolled in the NBS		4.08	0.81	1332G > A	K444=	1332G > A	K444=	Severe
S-02	Not enrolled in the NBS		5.11	2.59	652G > A	E218K	652G > A	E218K	Severe
S-03	Not enrolled in the NBS		3.46	2.75	538G > A	A180T	603C > A	Y201*	Hypoglycemic
S-04	Not enrolled in the NBS		0.97	3.07	796C > G<br>1153C > T	P266A<br>R385W	1269G > A	S423=	Hypoglycemic
S-05	Not enrolled in the NBS		6.49	5.55	996dupT	A333Cfs	1144A > C	K382Q	Hypoglycemic
S-06	Not enrolled in the NBS		2.62	9.75	520G > A	V174M	1349G > A	R450H	Hypoglycemic
S-07	Not enrolled in the NBS		0.82	6.41	996dupT	A333Cfs	1639G > A	V547M	Myopathic
S-08	Not enrolled in the NBS		0.84	6.47	1332G > A	K444=	1349G > A	R450H	Myopathic
S-09	Not enrolled in the NBS		5.80	6.60	514C > G	L172V	623-18_623-16delTAG		Myopathic
S-10	Not enrolled in the NBS		3.72	6.95	796C > G	P266A	1153C > T	R385W	Myopathic
S-11	Not enrolled in the NBS		6.11	7.66	906C > G	I302M	1246G > A	A416T	Myopathic
S-12	Not enrolled in the NBS		0.69	12.16	790A > G	K264E	1793A > G	H598R	Myopathic
S-13	Not enrolled in the NBS		3.66	12.39	833_835delAGA	K278del	1349G > A	R450H	Myopathic
S-14	Not enrolled in the NBS		1.75	13.72	790A > G	K264E	1048G > A	G350S	Myopathic

Abbreviations: NBS, newborn screening; VLCAD, very-long-chain acyl-CoA dehydrogenase; ND, no data
[1] Mean reference VLCAD activity in 54 normal control adults is 149.9 ± 57.1 pmol/min/10^6 lymphocytes.
[2] Most of the variants have been described in a previous report [18].
