# Supplementary material for: Using the C14:1/Medium-Chain Acylcarnitine Ratio Instead of C14:1 to Reduce False-Positive Results for Very-Long-Chain Acyl-CoA Dehydrogenase Deficiency in Newborn Screening in Japan
Source: Int J Neonatal Screen. 2024 Feb 20;10(1):15. doi: 10.3390/ijns10010015 (PMC10885094; doi:10.3390/ijns10010015)
Supplement: Supplementary file 1 [file IJNS-10-00015-s001.zip › TableS2.pdf]

Table S2. Concentrations of acylcarnitines and free carnitine in dried blood specimens of newborns.

Case ID	Acylcarnitine Levels in Dried Blood Specimens of Newborns (nmol/mL)													
	C18:1	C18	C16	C16-OH	C14:1	C14	C12	C10	C8	C6	C4	C3	C2	C0
N-01	ND	1.61	4.71	0.04	3.38	1.70	ND	0.168	0.076	ND	ND	0.84	11.97	21.57
N-02	ND	0.92	2.24	0.016	1.26	ND	ND	0.067	0.046	ND	ND	1.09	9.24	17.16
N-03	3.62	3.81	12.70	0.051	4.29	4.24	0.70	0.204	0.068	ND	ND	1.24	11.370	36.97
N-04	0.71	0.55	1.72	0.01	0.78	0.42	0.35	0.12	0.06	0.03	0.12	0.94	12.43	12.82
N-05	5.28	3.16	9.06	0.09	5.65	4.28	1.07	0.30	0.11	0.06	0.17	0.72	13.96	25.98
N-06	1.43	0.81	2.50	0.02	0.96	0.63	0.66	0.18	0.08	0.04	0.22	2.31	21.70	23.80
N-07	ND	1.15	2.62	0.024	0.65	ND	ND	0.164	0.081	ND	ND	1.38	13.36	27.24
N-08	2.32	0.80	2.56	0.012	0.42	0.26	0.18	0.077	0.084	0.018	0.10	1.93	19.20	13.00
N-09	2.05	1.21	3.02	0.02	2.80	ND	0.55	0.20	0.13	ND	ND	0.79	6.04	22.39
N-10	1.97	1.39	3.63	0.03	2.23	1.11	0.55	0.18	0.07	0.04	ND	2.59	25.04	24.91
N-11	2.59	2.25	5.28	0.04	2.73	1.96	0.70	0.13	0.07	0.06	0.18	1.43	19.10	26.35
N-12	1.07	0.86	2.31	0.02	0.40	0.28	0.23	0.11	0.07	0.03	0.14	1.36	12.16	18.39
N-13	1.43	0.83	1.88	0.018	0.40	0.34	0.32	0.15	0.07	0.02	0.12	1.82	16.70	22.08
N-14	2.32	1.34	4.62	0.056	4.79	1.85	1.21	0.342	0.175	0.076	0.21	1.98	32.40	22.50
N-15	1.47	0.99	2.07	0.01	0.61	0.30	ND	0.061	0.037	ND	ND	0.84	12.02	27.39
N-16	1.75	0.91	2.20	0.01	0.90	0.54	0.27	0.07	0.05	0.03	0.14	1.55	12.95	24.62
N-17	0.97	0.41	0.86	0.02	0.46	0.23	0.16	0.06	0.04	ND	ND	1.21	11.20	18.70
N-18	2.47	2.67	4.88	0.048	2.21	1.63	0.49	0.09	0.06	0.04	0.09	0.69	19.88	24.00
N-19	1.84	0.94	2.56	0.02	0.42	0.30	0.26	0.12	0.06	0.03	0.16	1.70	18.90	23.00
N-20	1.39	0.73	2.04	0.01	1.08	0.46	0.36	0.12	0.07	0.05	0.20	1.57	9.99	20.06
N-21	1.39	1.18	2.58	0.025	1.17	0.61	0.43	0.121	0.084	ND	ND	1.73	15.89	31.67
N-22	0.81	0.62	0.93	0.01	0.31	0.19	0.14	0.04	0.04	0.02	0.09	0.61	4.28	12.51
N-23	1.75	1.13	2.56	0.02	0.68	0.31	0.30	0.133	0.087	0.046	0.25	2.25	25.80	23.10
N-24	1.36	0.89	2.89	0.028	1.37	0.82	0.41	0.165	0.065	0.04	0.155	1.96	19.52	19.21
N-25	1.01	0.68	1.60	0.02	0.33	0.22	0.19	0.08	0.06	0.03	0.14	1.44	10.87	20.23
N-26	1.71	1.29	2.76	0.027	1.16	0.57	0.40	0.13	0.09	0.07	0.15	0.94	20.85	22.25
N-27	0.84	0.64	1.92	0.014	0.37	0.29	0.22	0.08	0.04	0.02	ND	1.30	14.55	13.12
N-28	1.74	1.14	4.19	0.027	1.31	ND	ND	ND	0.05	0.03	0.10	ND	10.31	ND
N-29	1.19	0.80	2.00	0.02	0.61	0.40	0.33	0.13	0.08	0.04	0.16	1.08	11.21	17.69
N-30	1.82	1.54	2.86	0.02	0.95	0.53	0.28	0.06	0.03	0.03	0.15	1.05	13.65	26.89
N-31	1.07	0.80	2.98	0.02	0.97	0.57	0.26	0.10	0.05	0.04	0.14	1.43	16.55	22.40
N-32	1.78	0.98	3.28	0.018	1.08	0.60	0.31	0.12	0.05	0.03	0.20	2.02	17.31	25.16
N-33	0.76	0.58	1.87	0.013	0.46	0.31	0.20	0.08	0.04	0.02	0.11	0.86	8.42	20.05
N-34	1.64	1.02	4.00	0.041	1.37	0.80	0.86	0.362	0.184	0.063	0.211	1.50	38.25	21.43
N-35	1.51	0.72	2.10	0.01	0.52	0.36	0.31	0.12	0.08	0.03	0.11	1.60	14.20	18.10
N-36	1.52	0.80	2.29	0.01	0.48	0.37	0.25	0.07	0.04	ND	ND	1.30	14.25	22.97
N-37	1.18	1.13	3.67	0.024	0.57	0.44	0.45	0.206	0.116	0.038	0.145	2.28	18.88	25.95
N-38	1.95	0.89	3.09	0.02	1.03	0.64	0.64	0.21	0.12	0.04	0.16	1.59	15.20	19.60
N-39	1.65	1.10	3.38	0.04	0.67	0.47	0.36	0.14	0.10	0.07	0.18	2.19	16.21	20.91
N-40	ND	1.09	3.39	0.032	1.24	ND	ND	0.102	0.079	ND	ND	1.01	12.29	21.44
N-41	0.74	0.59	1.48	0.011	0.25	0.16	0.09	0.06	0.03	0.02	0.09	0.66	7.16	12.98
N-42	1.70	0.89	2.62	0.015	0.40	0.34	0.20	0.09	0.05	0.04	0.21	2.32	24.16	34.67
N-43	1.33	1.00	1.98	0.01	0.44	0.26	0.15	0.03	0.02	ND	ND	0.95	5.62	18.30
N-44	1.22	0.90	2.35	0.02	0.48	0.41	0.24	0.12	0.09	0.03	0.13	1.17	13.39	19.37

N-45	2.92	2.91	9.54	0.10	4.57	4.29	0.72	0.34	0.11	0.13	0.09	0.73	12.40	20.10
N-46	ND	0.74	2.36	0.015	0.27	0.24	0.21	0.072	0.035	0.018	0.136	2.13	11.03	18.80
N-47	1.42	0.78	2.16	0.011	0.48	0.34	0.24	0.13	0.05	0.02	0.09	1.18	11.50	15.90
N-48	1.34	0.95	3.09	0.032	1.15	0.58	0.68	0.454	0.182	0.059	0.152	0.95	21.86	16.39
N-49	ND	ND	4.15	0.07	1.76	1.06	1.16	0.34	0.29	0.13	0.08	1.66	19.30	21.20
N-50	1.17	0.70	2.57	0.017	0.46	ND	ND	ND	0.06	ND	ND	ND	21.90	ND
N-51	ND	1.48	4.90	0.06	0.90	ND	ND	0.25	0.13	ND	ND	1.38	23.45	19.89
N-52	1.10	0.56	1.71	0.018	0.46	0.23	0.24	0.14	0.074	0.032	0.105	1.90	13.86	19.97
N-53	1.31	0.86	1.92	0.02	0.41	0.25	0.24	0.13	0.06	0.04	0.12	1.41	15.59	23.15
N-54	1.70	0.88	2.67	0.015	0.37	0.36	0.25	0.088	0.049	ND	ND	1.77	14.78	21.45
N-55	1.72	1.13	3.32	0.02	1.05	0.73	0.48	0.10	0.06	ND	ND	0.97	16.22	27.38
N-56	0.99	1.14	1.74	UD	0.35	0.20	0.17	0.05	0.03	0.02	0.10	0.98	6.69	24.84
N-57	1.62	1.05	3.58	0.029	0.74	0.40	0.40	0.21	0.10	0.04	0.15	0.74	14.07	24.10
N-58	1.28	0.89	2.45	0.02	0.61	0.36	0.31	0.10	0.06	0.04	0.19	0.98	8.64	14.82
N-59	1.60	0.95	2.88	0.015	0.33	0.40	0.19	0.06	0.04	0.03	0.15	2.38	17.21	32.34
N-60	1.74	1.01	3.70	0.03	0.46	0.39	ND	0.189	0.11	ND	ND	1.40	23.77	14.13
N-61	1.29	1.05	2.60	0.02	0.42	0.40	0.29	0.10	0.05	0.02	0.13	1.67	15.38	15.64
N-62	1.61	1.04	3.26	0.031	1.59	0.67	0.66	0.20	0.08	0.04	0.21	1.58	27.28	24.70
N-63	1.53	0.90	2.17	0.01	0.53	0.31	0.24	0.09	0.04	0.03	0.13	1.64	11.50	21.60
N-64	1.00	0.70	1.77	0.01	0.42	0.29	0.24	0.11	0.08	0.03	0.14	1.01	10.83	16.17
N-65	1.24	0.58	1.46	0.02	1.53	0.53	0.71	0.34	0.15	0.05	0.15	1.14	13.80	22.00
N-66	1.88	1.04	3.79	0.03	1.51	0.84	0.59	0.14	0.07	0.04	0.21	1.52	22.08	21.16
N-67	1.20	0.80	1.43	0.01	0.31	0.19	0.17	0.09	0.04	0.03	0.17	0.90	9.11	9.90
N-68	ND	0.97	3.13	0.03	1.00	0.70	0.37	0.208	0.11	ND	ND	1.28	20.72	36.15
N-69	0.96	0.74	1.58	0.02	0.30	0.21	0.20	0.10	0.08	0.03	0.25	1.20	11.53	15.49
N-70	0.69	0.41	1.29	0.01	0.44	0.22	0.21	0.08	0.05	ND	ND	1.29	12.18	15.91
N-71	1.53	0.86	3.29	0.023	0.51	0.43	0.39	0.20	0.067	0.042	0.373	1.97	12.77	24.28
N-72	1.22	0.92	2.37	0.014	0.40	0.27	0.21	0.16	0.07	0.04	0.11	1.42	14.92	17.83
N-73	1.57	1.31	3.35	0.018	0.40	0.37	0.18	0.10	0.03	0.02	0.14	1.86	21.15	23.95
N-74	0.76	0.47	1.51	0.014	0.43	0.24	0.25	0.099	0.05	ND	ND	0.96	9.75	15.82
N-75	1.78	1.25	3.23	0.028	1.81	0.97	0.44	0.14	0.08	0.04	0.32	2.44	16.20	26.34
N-76	1.07	0.88	1.98	0.017	0.35	0.36	0.27	0.16	0.06	0.03	0.14	0.79	15.34	15.34
N-77	1.44	0.80	2.81	0.01	0.46	ND	0.33	0.20	0.10	0.03	ND	2.33	18.55	13.85
N-78	1.35	0.83	1.74	0.012	0.30	0.25	0.15	0.10	0.04	0.03	0.13	1.71	12.84	23.20
N-79	0.99	0.55	1.49	0.012	0.39	0.24	0.26	0.39	0.08	0.03	0.17	1.50	8.75	13.90
N-80	1.83	1.27	3.91	0.018	0.41	0.42	0.23	0.079	0.052	0.04	0.121	1.52	21.21	22.57
N-81	0.97	0.60	1.93	0.02	0.45	0.30	0.41	0.16	0.09	0.04	0.06	0.96	14.51	8.89
N-82	1.50	0.95	2.18	0.017	0.40	0.32	0.15	0.06	0.03	0.02	0.11	1.17	11.35	13.60
N-83	1.83	0.93	2.27	0.012	0.39	0.25	0.21	0.16	0.07	0.03	0.22	2.42	19.39	27.14
N-84	0.95	0.69	2.22	0.014	0.48	0.31	0.23	0.13	0.06	0.03	0.20	1.26	15.63	13.70
N-85	0.81	0.53	1.50	0.02	0.45	0.30	0.24	0.10	0.07	0.03	0.14	1.38	13.63	15.11
N-86	ND	1.57	5.66	0.03	0.63	0.56	0.42	0.158	0.086	ND	ND	1.80	22.57	21.28
N-87	1.31	0.62	2.48	0.02	0.44	0.33	0.24	0.14	0.10	0.03	0.31	2.07	11.35	16.42
N-88	1.66	0.79	5.17	0.049	0.62	0.59	0.49	0.155	0.089	0.037	0.124	1.54	21.83	28.62
N-89	1.15	1.05	2.62	0.028	0.71	0.49	0.42	0.174	0.105	0.063	0.208	1.16	18.68	25.18
N-90	1.87	1.32	3.07	0.02	0.89	0.49	0.37	0.15	0.09	0.05	0.38	1.74	13.82	17.15
N-91	ND	ND	ND	0.03	0.66	ND	ND	0.119	0.08	ND	ND	1.19	20.56	ND

N-92	1.88	0.86	2.93	0.023	0.56	0.40	0.34	0.181	0.109	ND	ND	1.82	24.22	18.58
N-93	1.16	0.67	2.66	0.017	0.42	0.25	0.41	0.20	0.102	0.038	0.143	0.83	16.60	n9.48
N-94	1.66	1.17	3.19	0.02	0.80	0.49	0.41	0.13	0.07	0.04	0.20	1.19	17.81	15.70
N-95	1.58	0.83	3.01	0.019	0.47	0.28	0.41	0.32	0.16	0.08	0.23	2.28	23.54	22.53
N-96	1.30	0.46	3.08	0.02	0.41	ND	ND	0.31	0.16	ND	ND	1.27	20.83	26.80
N-97	1.32	0.61	2.50	0.022	0.30	0.25	0.29	0.09	0.09	0.03	0.15	1.20	16.08	15.46
N-98	1.37	0.85	3.71	0.04	0.60	0.55	0.51	0.21	0.09	0.03	0.19	1.96	22.30	15.13
N-99	1.70	1.03	4.35	0.031	0.59	0.52	0.47	0.305	0.156	0.068	0.158	1.56	33.95	30.34
N-100	1.03	0.96	2.27	0.023	0.24	0.21	0.20	0.12	0.08	0.03	0.13	0.98	13.95	11.80
N-101	0.90	0.67	1.82	0.01	0.20	0.19	0.12	0.10	0.05	0.02	0.13	1.13	8.80	10.10
N-102	1.63	1.04	3.06	0.018	0.40	0.27	0.20	0.074	0.042	0.037	0.132	1.33	17.22	25.04
N-103	1.77	1.03	3.17	0.021	0.36	0.28	0.30	0.20	0.12	0.05	0.23	2.07	30.04	39.32
N-104	1.58	0.96	2.81	0.018	0.48	0.31	0.37	0.26	0.16	0.06	0.026	2.08	16.30	21.60
N-105	1.30	ND	3.18	0.031	0.98	ND	0.54	0.236	0.097	0.032	0.211	1.62	16.64	18.90
N-106	1.75	0.96	3.66	0.05	1.28	0.76	1.00	0.37	0.19	0.11	ND	1.27	21.57	15.51
N-107	1.34	0.80	2.98	0.024	0.39	0.21	0.29	0.17	0.10	0.07	ND	0.95	23.06	21.62
N-108	1.36	0.66	2.40	0.02	0.43	0.31	0.45	0.45	0.19	0.05	0.20	1.59	18.70	21.10
N-109	1.94	1.26	3.63	0.03	0.31	0.34	0.23	0.12	0.09	0.06	0.42	1.59	19.19	11.59
N-110	0.88	0.82	3.10	0.011	0.27	0.31	0.24	0.19	0.11	0.05	0.25	1.29	17.70	13.90
N-111	1.38	0.64	2.38	0.02	0.44	ND	0.48	0.37	0.18	ND	ND	0.94	13.42	18.44
N-112	1.09	1.01	3.27	0.028	0.34	0.32	0.31	0.26	0.09	0.03	0.16	0.80	19.50	13.70
N-113	1.17	0.67	2.88	0.019	0.40	0.37	0.27	0.14	0.07	0.04	0.24	1.23	16.45	17.08
N-114	1.21	0.65	2.67	0.02	0.56	0.32	0.53	0.38	0.15	0.03	0.20	1.03	16.87	18.69
N-115	2.09	1.23	5.92	0.051	0.78	0.80	0.57	0.25	0.10	0.04	0.38	2.46	25.15	17.59
N-116	1.08	0.66	2.79	0.03	0.31	0.29	0.26	0.29	0.15	0.05	0.15	0.93	18.13	16.49
N-117	0.96	0.65	3.12	0.026	0.40	0.33	0.38	0.22	0.11	0.04	0.25	1.57	15.58	17.33
N-118	1.30	0.74	2.74	0.02	0.54	0.31	0.35	0.17	0.08	0.04	0.19	2.39	18.08	20.53
N-119	1.92	2.10	4.90	UD	0.60	ND	0.60	0.60	0.30	0.07	ND	1.50	31.00	14.00
N-120	1.43	0.60	2.88	0.02	0.42	0.35	0.39	0.23	0.11	ND	ND	1.78	18.70	12.98
N-121	1.36	0.88	2.73	0.035	0.43	0.30	0.38	0.27	0.14	0.05	0.22	1.36	15.20	8.15
N-122	1.01	0.74	2.84	0.04	0.71	0.42	0.72	0.44	0.24	0.12	0.22	0.73	18.56	9.71
N-123	1.64	1.00	4.29	0.044	0.68	0.45	0.73	0.477	0.207	0.045	0.275	2.32	28.23	19.30
N-124	1.00	0.55	4.41	0.012	0.41	0.30	0.42	0.30	0.13	ND	ND	1.64	21.83	14.09
N-125	1.61	0.90	4.35	0.02	0.33	0.34	0.31	0.18	0.11	0.03	0.25	2.07	27.00	17.24
N-126	1.58	0.79	2.65	0.015	0.32	0.27	0.29	0.25	0.09	0.03	0.16	1.08	12.93	14.30
N-127	1.51	0.96	3.88	0.037	0.35	0.41	0.35	0.20	0.10	0.06	0.19	0.84	21.46	8.69
N-128	1.39	0.84	2.64	0.01	0.30	0.23	0.23	0.26	0.13	0.08	0.32	1.63	15.21	26.77
N-129	1.48	0.86	3.33	0.02	0.28	0.30	0.25	0.14	0.11	ND	ND	1.00	18.59	11.43
N-130	1.30	0.68	1.97	0.011	0.20	0.17	0.15	0.20	0.08	0.02	0.11	0.83	9.90	12.68
N-131	ND	0.60	2.23	0.02	0.56	0.38	0.49	0.33	0.15	ND	ND	1.33	18.08	12.55
N-132	1.73	0.95	3.75	0.02	0.55	0.40	0.31	0.24	0.08	0.03	0.14	2.30	28.63	21.61
N-133	1.28	0.65	2.69	0.019	0.43	0.33	0.47	0.36	0.16	0.07	0.18	1.15	26.57	15.36
N-134	ND	ND	ND	0.02	0.37	ND	ND	0.184	0.09	ND	ND	2.00	17.14	26.79
N-135	ND	0.57	3.05	0.033	0.28	0.27	0.30	0.27	0.21	ND	ND	1.22	33.18	9.01
N-136	1.22	0.68	2.88	0.04	0.40	0.24	0.48	0.372	0.146	0.06	0.132	0.62	18.01	8.73
N-137	1.97	1.17	4.33	0.04	0.38	0.26	0.38	0.30	0.116	0.047	0.235	1.03	26.44	15.62
N-138	1.61	0.83	3.83	0.029	0.35	0.36	0.36	0.29	0.17	ND	ND	2.01	36.08	15.65

N-139	1.81	0.72	3.23	0.044	0.43	0.30	0.24	0.29	0.21	0.08	0.31	2.32	31.45	13.12
N-140	1.34	0.70	2.76	0.035	0.41	0.29	0.39	0.322	0.18	0.088	0.23	1.21	23.50	23.20
N-141	1.06	0.52	2.30	0.026	0.42	0.26	0.47	0.348	0.173	0.074	0.254	1.51	22.59	15.12
N-142	1.59	0.77	3.23	0.018	0.34	0.25	0.33	0.32	0.21	0.10	0.18	1.02	29.41	16.50
N-143	1.36	0.91	3.13	0.022	0.30	0.30	0.25	0.19	0.11	0.04	0.23	2.17	13.34	21.82
N-144	1.50	0.70	3.70	0.045	0.46	0.33	0.37	0.38	0.17	0.09	0.22	1.22	19.24	13.32
N-145	1.70	0.82	3.99	0.034	0.30	0.31	0.33	0.44	0.23	0.09	0.2	1.57	19.42	13.00
N-146	1.40	0.67	2.88	0.028	0.32	0.24	0.34	0.38	0.20	0.08	0.21	1.82	19.08	11.25
N-147	1.72	0.62	3.08	0.04	0.47	0.29	0.32	0.36	0.20	0.08	0.12	1.42	21.28	11.16
N-148	1.49	0.80	3.10	0.037	0.30	0.25	0.26	0.33	0.14	0.10	0.28	1.82	24.24	15.72
N-149	1.40	0.73	3.80	0.045	0.33	0.28	0.27	0.32	0.16	0.06	0.20	1.14	21.86	17.73
N-150	1.41	0.48	2.61	0.019	0.33	0.30	0.33	0.26	0.13	0.07	0.18	1.10	22.29	15.99
N-151	1.54	1.11	3.60	0.033	0.32	0.28	0.32	0.40	0.16	0.09	0.30	1.16	25.00	13.82
N-152	1.56	0.85	2.94	0.026	0.31	0.35	0.29	0.33	0.16	0.09	0.25	1.82	26.24	13.05
N-153	1.90	0.85	3.88	0.044	0.35	0.30	0.40	0.25	0.11	0.06	0.22	0.92	29.39	25.21
N-154	1.15	0.84	3.41	0.036	0.31	0.31	0.39	0.26	0.12	0.06	0.20	1.47	22.40	11.33
N-155	1.27	0.60	2.63	0.038	0.40	0.26	0.28	0.33	0.19	0.09	0.26	1.08	22.05	10.64
N-156	1.77	0.94	4.01	0.045	0.41	0.42	0.35	0.35	0.20	0.10	0.27	1.21	28.95	13.82
N-157	1.17	0.59	2.81	0.059	0.37	0.30	0.33	0.22	0.12	0.067	0.35	0.80	15.20	8.70
N-158	1.84	1.05	3.85	0.037	0.35	0.30	0.38	0.59	0.27	0.11	0.26	1.02	24.45	22.36
N-159	1.87	1.09	4.14	0.041	0.40	0.41	0.30	0.28	0.19	0.08	0.31	1.47	25.72	21.32
N-160	ND	0.61	4.02	0.019	0.31	0.25	0.36	0.42	0.18	ND	ND	1.33	29.73	15.65
N-161	1.83	0.96	3.40	0.034	0.32	0.29	0.28	0.25	0.12	0.08	0.35	2.86	26.41	16.68
N-162	0.98	0.56	2.36	0.017	0.30	0.21	0.36	0.33	0.19	0.10	0.59	1.04	18.19	14.99
N-163	1.54	1.14	4.04	0.039	0.34	0.42	0.37	0.25	0.14	0.06	0.23	1.18	22.70	10.27
N-164	1.31	0.71	2.46	0.018	0.31	0.19	0.32	0.28	0.14	0.05	0.20	0.85	15.42	15.92
N-165	1.55	0.70	3.54	0.032	0.38	0.29	0.34	0.55	0.25	0.10	0.41	1.30	27.97	13.22
N-166	1.54	0.559	3.45	0.065	0.34	0.26	0.32	0.473	0.174	0.077	0.126	0.89	17.69	9.04
N-167	1.58	1.00	3.51	0.04	0.40	0.36	0.42	0.64	0.28	0.12	0.23	1.03	26.60	24.20
N-168	1.72	0.69	3.14	0.036	0.33	0.34	0.40	0.56	0.23	0.09	0.27	1.70	27.74	18.37
N-169	1.71	0.92	4.35	0.037	0.37	0.33	0.31	0.26	0.10	0.06	0.22	1.52	31.08	26.22
N-170	2.68	1.49	5.66	0.058	0.37	0.57	0.39	0.38	0.18	0.10	0.35	2.16	29.56	17.21
N-171	1.97	1.08	4.44	0.026	0.35	0.40	0.36	0.35	0.19	ND	ND	1.59	34.44	14.25
N-172	1.83	1.14	4.15	0.031	0.37	0.38	0.36	0.29	0.16	0.05	0.19	1.13	23.41	9.69
N-173	1.58	1.00	4.57	0.083	0.39	0.42	0.31	0.29	0.15	0.06	0.40	2.83	32.19	21.38
N-174	1.36	0.56	2.98	0.033	0.31	0.26	0.33	0.40	0.21	0.13	0.20	0.95	25.73	18.73
N-175	2.03	1.34	5.38	0.044	0.35	0.47	0.37	0.34	0.15	0.07	0.30	2.23	31.84	21.00

Abbreviations: C0, free carnitine; ND, no data; UD, undetectable
